# Super-resolution re-scan second harmonic generation microscopy

**DOI:** 10.1073/pnas.2214662119

**Published:** 2022-11-14

**Authors:** Stefan G. Stanciu, Radu Hristu, George A. Stanciu, Denis E. Tranca, Lucian Eftimie, Adrian Dumitru, Mariana Costache, Harald A. Stenmark, Harm Manders, Amit Cherian, Mariliis Tark-Dame, Erik M. M. Manders

**Affiliations:** ^a^Center for Microscopy-Microanalysis and Information Processing, University Politehnica of Bucharest, 060042 Bucharest, Romania;; ^b^Pathology Department, Emergency Military Hospital, 010825 Bucharest, Romania;; ^c^Department of Pathology, Bucharest Emergency University Hospital, 050098 Bucharest, Romania;; ^d^Department of Pathology, Carol Davila University of Medicine and Pharmacy, 050474 Bucharest, Romania;; ^e^Department of Molecular Cell Biology, Institute for Cancer Research, Oslo University Hospital, 0379 Oslo, Norway;; ^f^Confocal.nl BV, Science Park 406, 1098XG Amsterdam, The Netherlands

**Keywords:** second harmonic generation, two-photon excited fluorescence, super-resolved optical imaging

## Abstract

Second harmonic generation microscopy (SHG) is generally acknowledged as a powerful tool for the label-free three-dimensional visualization of tissues and advanced materials, with one of its most popular applications being collagen imaging. Despite the great need, progress in super-resolved SHG imaging lags behind the developments reported over the past years in fluorescence-based optical nanoscopy. In this work, we demonstrate super-resolved re-scan SHG, qualitatively and quantitatively showing on collagenous tissues the available resolution advantage over the diffraction limit. We introduce as well super-resolved re-scan two-photon excited fluorescence microscopy, an imaging modality not explored to date.

Second harmonic generation microscopy(SHG) exploits the interaction of two incident near-infrared photons with a noncentrosymmetric molecule, resulting in a single emitted photon with double the energy via a nonlinear process involving virtual states. Considering its high imaging potential based on intrinsic contrast, augmented by other advantages such as no bleaching, no blinking, or high signal-to-noise ratio compared to fluorescence, SHG has gained important interest in the context of label-free characterization of fixed, ex vivo and in vivo tissues (1–3) and advanced materials (4, 5). With respect to the former, selective SHG contrast from collagen holds valuable potential for disease diagnostics, given that during the progression of many severe pathologies, including cancers, the collagen network of affected organs is modified in specific ways (6). Applications based on SHG contrast from exogenous harmonophores are also noteworthy to mention (7). In materials science, among others, SHG has been demonstrated useful for characterizing defects in semiconductors (8), carrier motion in organic transistors (9), grain boundaries for two-dimensional materials (4), or for the overall better understanding of emerging materials (5).

Among the first notable attempts to achieve super-resolved images based on SHG, Masihzadeh et al. (10) and Liu et al. (11) succeeded in reducing the size of the point-spread function by manipulating the polarization state of the incident light. A drawback of such approaches is their high sensitivity to effects such as circular dichroism or birefringence, which interfere with the polarization state of the excitation beam. Later, Field et al. (12) introduced multiphoton spatial frequency-modulated imaging, a technique with no specific polarization requirements, providing a 2× resolution improvement over conventional SHG and two-photon excited fluorescence (TPEF); the complex experimental setup might raise difficulties for non-expert users. Further on, Gregor et al. (13) achieved super-resolved SHG and TPEF images using image scanning microscopy (ISM). Under illumination with a 900-nm laser beam, distances of 550 nm with a contrast of 100% were successfully resolved for rat tail collagen fibrils. ISM uses a mathematic algorithm on many small pinhole images (sometimes over 10^6^) to reconstruct the improved resolution image, which has important implications with respect to the total acquisition time.

Herein we introduce re-scan second harmonic generation microscopy (rSHG), a method for super-resolved SHG imaging, which contributes to overcoming the aforementioned limitations. We demonstrate as well re-scan two-photon excited fluorescence (rTPEF), with TPEF generally regarded as a complementary non-linear optical microscopy (NLO) technique to SHG (3). Both rSHG and rTPEF rely on the re-scan concept which lies at the core of re-scan confocal microscopy (RCM) (14). Since its recent advent, RCM has gained massive interest and a large user base given its resolution advantages which are available under the premises of a simple and highly flexible technical architecture and affordable costs.

## Results

**Fig. 1.**
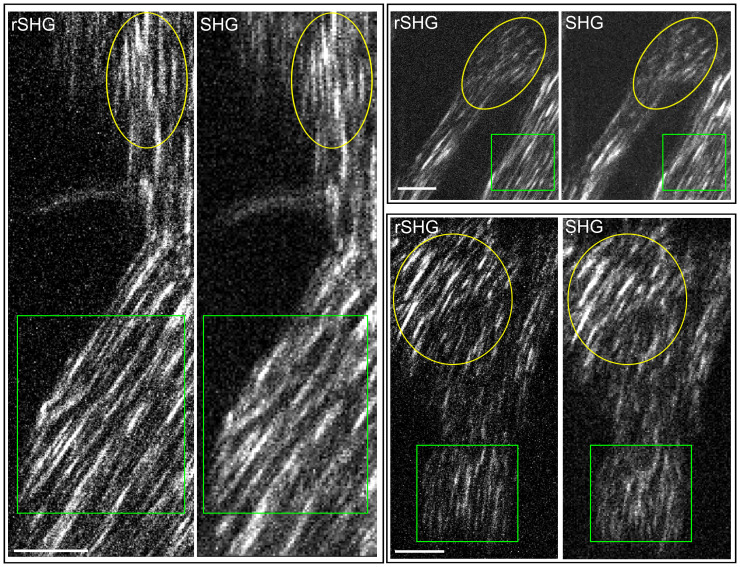
Resolution advantage of rSHG vs. SHG on rat tail collagenous tissues (formalin-fixed, unstained) at different levels of detail. Colored masks indicate sample areas where rSHG’s resolving power is well visible. Images collected with a scientific grade Hamamatsu ORCA-Flash 4.0 v3 s-CMOS camera. (Scale bars: 3 µm.)

**Fig. 2.**
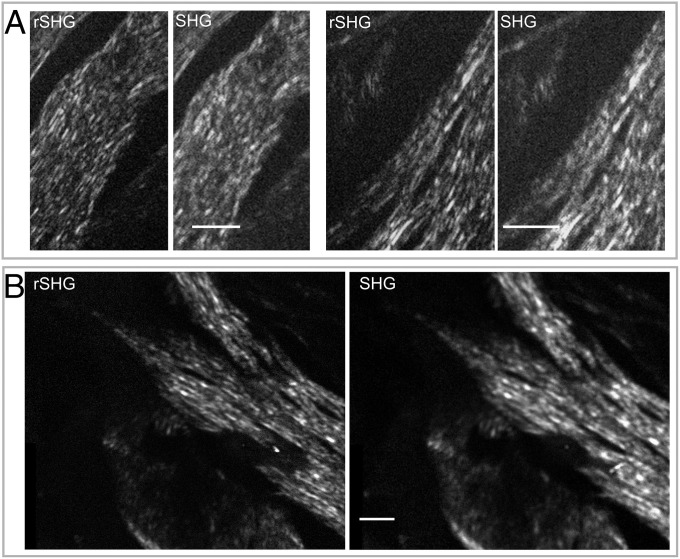
SHG and rSHG images of a tissue fragment (formalin-fixed, H&E-stained) from a negative resection margin adjacent to a breast carcinoma acquired with (*A*) a Hamamatsu ORCA-Flash 4.0 v3 scientific-grade sCMOS camera and (*B*) a FLIR Chameleon3 conventional CMOS camera. (Scale bars: 5 μm.)

For demonstrating the resolution advantage of rSHG we have used both a rat tail tissue fragment (fixed, unlabeled), a highly collagenous tissue that is emerging as a model for benchmarking SHG imaging methods, and also human breast tissue. In Figs. 1 and 2 we provide qualitative evidence on the resolution advantage of rSHG over conventional, diffraction-limited, SHG imaging. In Fig. 1, the colored masks indicate rat tail tissue areas where the collagen fibers and the overall aspect of the collagen network are significantly better resolved with rSHG. In Fig. 2 we present rSHG images collected on a hematoxylin and eosin (H&E)-stained normal breast tissue fragment, prepared according to typical procedures for clinical histopathological examination. As discussed in past work, H&E staining does not significantly influence SHG signals (15). rSHG allows assessing the conformation and disposition of the collagen in the connective stroma neighboring a healthy mammary terminal ductal–lobular unit with considerable better clarity than SHG.

**Fig. 3.**
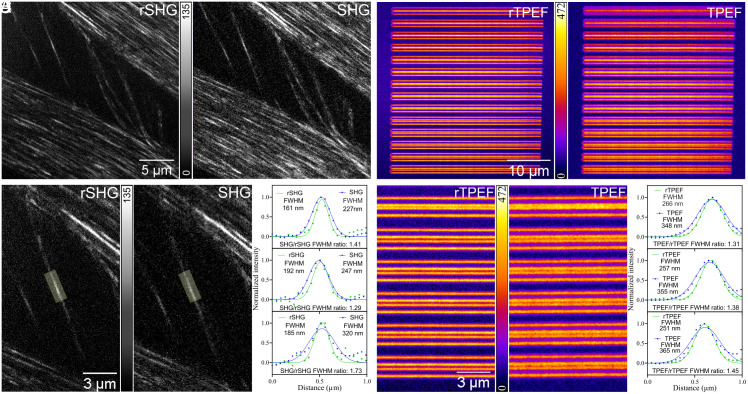
Resolution improvement in rSHG and rTPEF. (*A*) rSHG and SHG images collected on rat tail tendon collagen fibrils. (*B*) Examples of profile line pairs transversally drawn across the collagen fibril in the highlighted area. (*C*) rTPEF and TPEF images collected on an Argo-SIM calibration sample. (*D*) Examples of profile line pairs transversally drawn across the visible structures. Profile lines: green: rSHG/rTPEF, blue: SHG/TPEF.

In Fig. 3 we provide quantitative proof of the resolution enhancement provided by rSHG, and by rTPEF, with TPEF imaging being generally acknowledged as highly complementary to SHG (2, 3). All calculations discussed in this figure were performed on non-processed, non-averaged rSHG and rTPEF images. In Fig. 3*A* we present a rat tail tissue collagenous sample area where profile lines were drawn over well-separated collagen fibrils. In Fig. 3*B* we present a close-up view of a single collagen fibril, highlighting a region where the SHG and rSHG profile lines were drawn to quantitatively assess the resolution enhancement. The average full width at half maximum (FWHM) ratio for 10 profile lines randomly drawn over the highlighted area was 1.40 ± 0.2, with the average FWHM calculated for the rSHG and SHG images being 160 ± 16 nm and 220 ± 30 nm, respectively (results presented as mean ± SD). Similar values were identified for >70 considered profile lines drawn over single collagen fibrils in other acquired SHG and rSHG image pairs. rSHG and SHG images were collected with the same system (*SI Appendix*), with switching between the two work modes being done by modifying the angular amplitude of the scanner and re-scanner mirrors (*SI Appendix*). Noteworthy, the experimental FWHM value for the SHG image aligns to the theoretical value, which for 860-nm illumination and a 1.42 numerical aperture is 210 nm, according to Eq. **1**:

[1] $$\text{FWHM}=\frac{0.5\lambda }{\sqrt{2}\cdot \text{NA}}.$$

With the same setup we also explored rTPEF (Fig. 3*C*), which was performed on a fluorescent Argo-SIM (Argolight, France) calibration slide. For >50 profile lines pairs randomly considered in the displayed TPEF and rTPEF images, the average FWHM ratio was 1.51 ± 0.05, with the average FWHM calculated for the rTPEF and TPEF images being 241 ± 5 nm and 360 ± 10 nm, respectively. Examples of TPEF & rTPEF profile lines are provided in Fig. 3*D*.

## Discussion

The greater level of detail offered by rSHG over conventional SHG can obviously favor the more accurate extraction of quantitative information reflecting the morphology and other properties of the collagen fibrils (16, 17), which play an important role in computer-aided diagnostics scenarios. Furthermore, for methods aimed at differentiating pathological vs. healthy tissues based on pixel-by-pixel fitting of polarization-resolved SHG images of collagen fibrils (18), a higher optical resolution enables access to additional information which can augment the diagnostic procedure.

In terms of relevant applications, given that an RCM unit adapted for rSHG can be easily coupled to optical microscopes already available in clinical settings, we argue that histopathology laboratories can benefit from this reported methodology, as it can enable modern workflows based on NLO techniques to complement traditional assays. Such correlative approaches can be implemented on samples prepared using traditional histopathology protocols, as usual dyes (e.g., H&E) do not significantly affect the SHG signals. On the other hand, such stains have been shown to augment tissue imaging (19) with third harmonic generation microscopy (THG), which can potentially be implemented with the same re-scan architecture, depending on the availability of appropriate illumination sources. Furthermore, considering that super-resolved imaging of collagen, lipids, and various endogenous chromophores can also be performed on unstained tissues with rSHG, re-scan THG, and rTPEF, respectively, we argue that these techniques can be of important help to identify subtle modifications that precede, or develop, during cancers on freshly excised tissues, next to the patient. This can promote improved diagnostics and faster decision-making.

Overall, given the simplicity and flexibility of re-scan microscopy, we consider that the reported architecture and results are likely to augment the number and nature of applications relying on super-resolved non-linear optical imaging.

## Methods

rSHG and rTPEF were performed with a multimodal prototype based on a conventional RCM setup of first generation that was custom-modified. The architecture and configuration of this system, together with aspects on data acquisition and sample preparation, are presented in *SI Appendix*. The tissue fragments of human origin discussed in this work were previously collected for routine histological examination from a patient and were deidentified prior to use in the current study.

## Supplementary Material

Supplementary File

## Data Availability

Images used for quantitatively assessing the resolution advantage in rSHG/rTPEF, Fig. 3, have been deposited in Open Science Framework (OSF) (https://osf.io/2wgt5/) (20).
