# Supplementary material for: Super-resolution re-scan second harmonic generation microscopy
Source: Proc Natl Acad Sci U S A. 2022 Nov 14;119(47):e2214662119. doi: 10.1073/pnas.2214662119 (PMC9704717; doi:10.1073/pnas.2214662119)
Supplement: Supplementary File [file pnas.2214662119.sapp.pdf]

Supporting Information for

Super-resolution re-scan second harmonic generation microscopy

Stefan G. Stanciu,[1,‡,*] Radu Hristu,[1,‡] George A. Stanciu,[1] Denis E. Tranca,[1] Lucian Eftimie,[2] Adrian Dumitru,[3,4] Mariana Costache,[3,4] Harald A. Stenmark,[5] Harm Manders,[6] Amit Cherian,[6] Mariliis Tark-Dame,[6] and Erik M.M. Manders[6]

[1]Center for Microscopy-Microanalysis and Information Processing, University Politehnica of Bucharest, 060042 Bucharest, Romania

[2]Emergency Military Hospital, Pathology Department, 010825 Bucharest, Romania

[3]Department of Pathology, Bucharest Emergency University Hospital, 050098 Bucharest, Romania

[4]Department of Pathology, Carol Davila University of Medicine and Pharmacy, 050474 Bucharest, Romania

[5]Department of Molecular Cell Biology, Institute for Cancer Research, Oslo University Hospital, Montebello, 0379 Oslo, Norway

[6]Confocal.nl BV, Science Park 406, 1098XG Amsterdam, The Netherlands

‡These authors contributed equally to this work

*Corresponding author: Stefan G. Stanciu, e-mail: stefan.g.stanciu@upb.ro

This pdf file includes:

1. Optical setup of the multimodal prototype developed for rSHG/rTPEF imaging

2. Image acquisition and registration

3. Sample preparation

1. Optical setup of the multimodal prototype developed for rSHG/rTPEF imaging

rSHG and rTPEF were performed with a multimodal prototype based on a custom modified RCM setup of 1st generation. A schematic diagram of this system can be found in(1). To enable rSHG and rTPEF imaging, the RCM unit was equipped with a free-space laser input port. This port was used to introduce in the original RCM excitation path(2) the fs-beam originating from an infrared (IR) Chameleon Vision II (Coherent) Ti-Sapphire laser with ~140 fs pulses, a repetition rate of 80 MHz, tuned at 860 nm. A dichroic mirror ZT775sp-2p (Chroma) was used to combine the continuous wave (CW) beam(s) used for RCM imaging (405nm, 488nm, 561nm, 633nm) with the fs-laser beam used for rSHG (860nm), to enable multimodal RCM/rSHG/rTPEF imaging in specific applications that require combining these imaging modalities. By placing a mirror at the sample position, CW VIS light was "injected" on the fs IR arm. By adjusting the collinearity and overlap of the CW VIS and IR beams on the IR arm, outside the RCM case, allowed the alignment of the latter. The standard dichroic mirror and emission filter of a typical RCM unit have been kept in place, ZT405-488-561-640-NIR-rpc (Chroma) & ZET405-488-561-640m (Chroma), as they don't interfere with collecting the SHG/THG signals generated under 860nm fs IR beam excitation. Using the modified setup with other IR wavelengths for rSHG and rTPEF, may require removal of the emission and excitation filters used for standard RCM imaging. While the use of the pinhole is optional for rSHG and rTPEF, considering the intrinsic optical sectioning capabilities of these two techniques, removing it would interfere with the possibilities for multimodal RCM/rSHG/rTPEF imaging (not discussed here, in order not to divert focus). To avoid this, in the here reported setup, we used a pinhole that was kept fixed at 50 µm. Two additional short pass filters, ET750sp-2p8 (Chroma), were used to block the NIR excitation light. To extract SHG signals a band-pass filter ET430/24x (Chroma) was used, which was placed in a retractable 3D-printed filter holder, allowing switching between RCM, rSHG and rTPEF workmodes. For collecting the rTPEF images (on samples emitting no SHG signals) no band-pass filter was used.

The setup above described was coupled to a Ti2-E Nikon inverted microscope equipped with a 100x/1.42 NA objective. The rSHG and rTPEF images depicted in Fig. 1, Fig 2a) and Fig. 3 in the main text were acquired with scientific-grade CMOS Hamamatsu Flash 4.0 V3 camera, 2048 X 2048 pixels, with a unit cell size of 6.5 µm (H) × 6.5 µm (V), operated in a non-cooled configuration. The use of a standard

CMOS, Chameleon3 5.0 MP CMOS camera with 2448 X 2048 pixels SONY IMX264 sensor, with a unit cell size of 3.45 μm (H) × 3.45 μm (V), was also evaluated, Fig. 2b).

An important feature of an RCM unit stands in its provided facile possibilities for correlative imaging sessions with re-scan, brightfield and widefield fluorescence microscopy modalities. To this end, a system of retractable mirrors can be positioned on the optical path, so that the light emitted by the sample in brightfield microscopy and widefield fluorescence microscopy workmodes bypasses the scanner and the re-scanner (used for re-scan data acquisition) on its way to the camera. In 1st gen RCM units, this system of retractable mirrors can be easily inserted or removed from the optical path with the help of a knob. With the same objective, the field-of-view in brightfield and widefield fluorescence microscopies is higher compared to the field-of-view for re-scan workmodes, with the re-scan image being positioned within the borders of the brightfield/widefield fluorescence image (the exact position with respect to the center of the latter can be easily adjusted). In the case of the Hamamatsu Flash 4.0 V3 camera, the field-of-view contained in 1024 x 1024 pixels images collected with brightfield or widefield fluorescence microscopy and with re-scan modalities (RCM, rSHG, rTPEF) are 66.6 x 66.6 μm^2 and 44.4 x 44.4 μm^2, respectively.

2. Image acquisition and registration

In a typical RCM system, the CW laser is switched off during the return movement of the scanner to avoid ghosting effects (blur) that typically arise due to the mismatch in the angular speed of the scanner and rescanner, during their forward and backward movement. This leads to a phase difference of the scanner relative to the re-scanners. This problem is normally solved by hardware triggering, which synchronizes the on/off status of the laser with the forward/backward movement of the scanner. Implementing a similar strategy with the NIR fs-laser beam was not feasible as such high-frequency on/off modulation interferes with the pulse locking. Therefore, we used bi-directional scanning with a phase shift correction to minimize ghost image effects. Alternatively, a hardware-triggered Acusto-Optical Modulator positioned on the path of the NIR fs-laser beam can potentially be used to meet the same objective.

As thoroughly described in the work of De Luca et al.(2), in an RCM system a resolution improvement over the diffraction limit is achieved when the re-scan amplitude of the camera scanner (re-scanner) is higher than the angular amplitude of the sample scanner. In typical RCM implementations a ratio of 2

between the two angular amplitudes is used (Sweep factor [SF] = 2, in Confocal.nl RCM terminology). When the amplitudes of the two scanners match (SF = 1), the resolution yielded by the RCM is similar to the resolution of a conventional, diffraction limited, confocal laser scanning microscope. Fig. 1, 2 and 3 of the main text present rSHG images collected at SF = 1 and SF = 2, which we denoted as SHG and rSHG images, respectively. The rSHG images displayed in Fig. 1, 2a) and 3 were collected with the Hammamatsu Flash 4 v.3 camera, with 43nm pixel size, whereas the SHG images were collected with 86nm pixel size (a consequence of the SF setting). The rSHG and SHG images collected with the Chameleon 3 camera, Fig. 2b) have been collected with 23nm and 46 nm pixel size, respectively. Considering the difference in pixel size, to observe the same sample regions in both rSHG and SHG configurations, we used digital resolutions of 1024x1024 pixels and 512x512 pixels respectively. The 1024x1024 pixels rSHG images were collected with a speed of 200 Hz/line, and the same pixel dwell time was used for the SHG images. For qualitative assessment of the resolution advantage, we registered the SHG images to the rSHG ones by 2x upsampling (no interpolation). For the quantitative assessment part, all calculations and profile lines were measured on the original (non-registered and non-processed) images.

No image processing has been performed for any of the rSHG images collected with the scientific grade CMOS Hammamatsu camera present in this work (Fig. 1, Fig. 2a, Fig. 3). For the rSHG images collected with the standard CMOS Chameleon 3 camera (Fig. 2b), hot pixels were removed with the standard despeckle denoising option of Fiji(3). The rSHG images displayed in Fig. 2b) represent cropped regions of rSHG/SHG mosaics that were assembled from 2x2 image tiles with the MosaicJ(4) plugin of ImageJ(5).

3. Sample preparation

Preparation of the rat-tail tendon samples: The rat-tail tendon samples have been collected from Wistar rats that were sacrificed at the end of a different experiment, not discussed in this article, which was approved by the Ethics Committee of the National Institute for Medical-Military Research and Development "Cantacuzino" in Bucharest, Romania and also by The National Sanitary Veterinary and Food Safety Authority. These samples have been made available via a collaboration protocol following the 3R principle in Ethics to LE at the "Dr. Carol Davila" Central Military Emergency University Hospital. The imaged specimen samples were fixed with 10% buffered formalin for 24 hours and were processed by conventional

histopathological methods using paraffin embedding, 3 μm thick sectioning. These samples have not been stained.

Preparation of the H&E stained breast tissue samples: The tissue fragments discussed in this work were previously collected for routine histological exam from a patient diagnosed with moderately differentiated (G2) invasive breast cancer of no special type (NST) with extensive foci of high grade ductal carcinoma in situ (DCIS), using the current criteria from WHO Classification of Tumors of the Breast, 5th edition(6). The tumor grade was established using the Elston-Ellis (Nottingham score) grading system. The same samples that were previously used for clinical histological exam were imaged with rSHG/SHG (Fig. 2 of the main text). Specifically, rSHG was used to image normal breast tissue from healthy regions close to the resection margins of malignant/premalignant lesions. Patient informed consent on the use of collected samples for research purposes was acquired, and the use of histology samples from the archive of the Bucharest Emergency Hospital for research purposes is in line with the regulations of its Ethics Committee. The imaged specimen samples were fixed with 10% buffered formalin for 24 hours and were processed by conventional histopathological methods using paraffin embedding, 3 μm thick sectioning and Hematoxylin–Eosin (HE) staining.

References

1. S. G. Stanciu *et al.*, rSHG: Re-scan Second Harmonic Generation Microscopy. *arXiv preprint arXiv:2207.02002* (2022).
2. G. M. De Luca *et al.*, Re-scan confocal microscopy: scanning twice for better resolution. *Biomedical optics express* **4**, 2644-2656 (2013).
3. J. Schindelin *et al.*, Fiji: an open-source platform for biological-image analysis. *Nature methods* **9**, 676 (2012).
4. P. Thévenaz, M. Unser, User-friendly semiautomated assembly of accurate image mosaics in microscopy. *Microscopy research and technique* **70**, 135-146 (2007).
5. A. B. Schroeder *et al.*, The ImageJ ecosystem: Open-source software for image visualization, processing, and analysis. *Protein Science* **30**, 234-249 (2021).
6. L. Dilani, A. Valerie, W. Beiko (2019) WHO Classification of Tumours, Breast tumours. (Lyon: International Agency for Research on Cancer).
